# Progressive pseudorheumatoid dysplasia involving a novel *WISP3* mutation and sacroiliac and hip arthritis: A case report and literature review

**DOI:** 10.1097/MD.0000000000034099

**Published:** 2023-07-07

**Authors:** Weitao Wang, Guangzhi Xiao, Qing Han, Jin Ding, Ronghua Xie, Junfeng Jia, Nan Leng, Zhaohui Zheng

**Affiliations:** a Department of Clinical Immunology, Xijing Hospital, The Fourth Military Medical University, Xi’an, China.

**Keywords:** hip arthritis, progressive pseudorheumatoid dysplasia, sacroiliitis

## Abstract

Progressive pseudorheumatoid dysplasia (PPRD) is a rare autosomal recessive genetic disease caused by mutations in the *Wnt1-inducible signaling pathway protein 3* gene. PPRD is considered a noninflammatory disease, and involvement of the sacroiliac joint and hip arthritis have not been reported previously.

**Patient concerns:**

We report a case of PPRD in an 11-year-old boy, who presented with bilateral pain and swelling in the knees, elbows, and ankles, and bilateral pain without swelling in the shoulders, wrists, knuckles, and proximal and distal interphalangeal joints for the past 5 years. He had been misdiagnosed with juvenile idiopathic arthritis for more than 6 years.

**Diagnosis:**

The correct PPRD diagnosis was made using whole-exome sequencing for *Wnt1-inducible signaling pathway protein 3* gene mutations (c.589 + 2T>C and c.721T>G; both mutations have rarely been reported) and magnetic resonance imaging examination; moreover, the latter showed inflammation of the sacroiliac joint and hip joint.

**Intervention:**

The patient was administered supplemental calcium, active vitamin D, and glucosamine sulfate.

**Outcome:**

The patient experienced alleviation of joint pain following treatment initiation; however, joint motion improvement was not obvious. Above all, the long-term use of biologic or targeted synthetic disease-modifying antirheumatic drugs in the future was avoided.

**Conclusion:**

The findings of the inflammatory aspects in PPRD will enrich our understanding of this rheumatological disease.

## 1. Introduction

Progressive pseudorheumatoid dysplasia (PPRD) is caused by mutations in the *Wnt1-inducible signaling pathway protein 3 (WISP3*) gene. This gene is a member of the CYR61/CTGF/NOV family of growth factors that play an important role in skeletal development, angiogenesis, tumorigenesis, cell proliferation, adhesion, migration, and survival.^[1,2]^ PPRD is a rare childhood disease with an estimated incidence of 1/1000,000 in the United Kingdom.^[3]^ Most patients present with symptoms between 3 and 8 years of age, whereas some may be asymptomatic until the age of 16 years or older.^[2]^

As PPRD is rare, the available literature mainly includes single-case reports. More than 80 *WISP3* mutations (Table S1, Supplemental Digital Content, http://links.lww.com/MD/J178) have been reported^[1]^; however, their exact role remains unclear. Herein, we describe a case of PPRD with rarely reported *WISP3* mutations, c.589 + 2T>C and c.721T>G. Additionally, the patient had simultaneous sacroiliac and hip arthritis, which has not been previously reported.

## 2. Case presentation

An 11-year-old boy (height: 1.3 m; weight: 26 kg) was admitted with swelling in multiple joints and pain that had lasted for 5 years. At the age of 6 years, he developed bilateral pain and swelling in the knees, elbows, and ankles, and bilateral pain without swelling in the shoulders, wrists, knuckles, and proximal and distal interphalangeal joints. He did not experience fever, rash, nausea, vomiting, morning stiffness, hair loss, dry mouth, dry eyes, oral ulcers, or Raynaud phenomenon. He was first diagnosed with juvenile idiopathic arthritis (JIA). Additionally, he had previously been administered nonsteroidal anti-inflammatory drugs, methotrexate, and tumor necrosis factor-α inhibitors for a long time, although they were ineffective. His grandfather was diagnosed with Kashin–Beck disease, but neither of his parents were.

**Figure 1.**
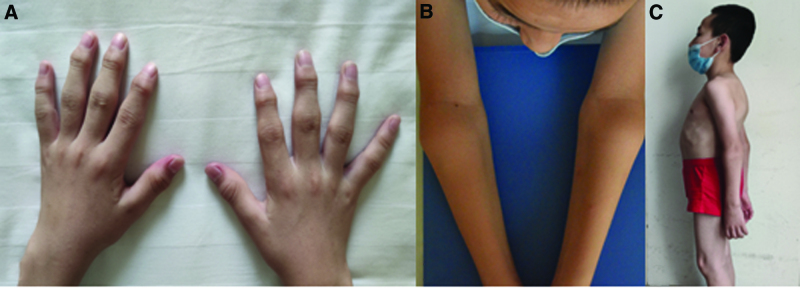
The physical presentation of the patient upon admission. (A) Obvious bilateral osseous enlargement of the interphalangeal joints, (B) swollen elbows, and (C) thoracic deformity.

**Figure 2.**
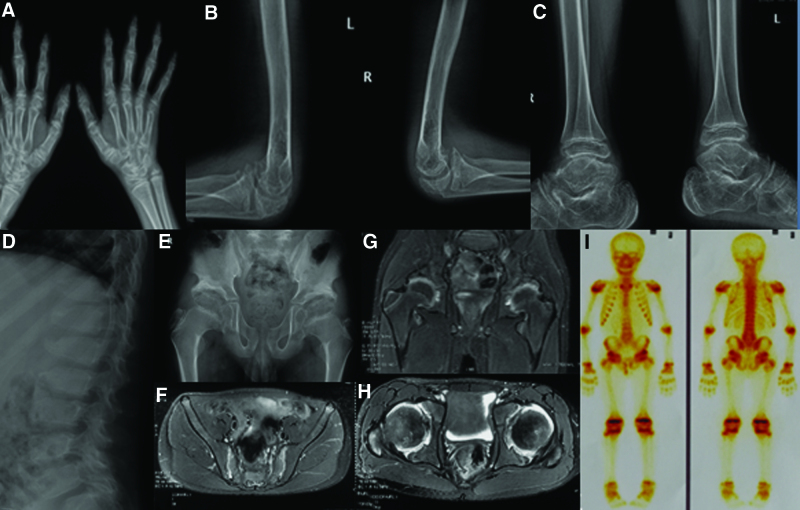
Imaging results of the patient. (A) X-ray of the hands shows dilatation of the interphalangeal joints; (B) elbows show underglazing of the articular surface and chondrodysplasia; (C) ankles show bilateral narrow joint spaces and incomplete joint surfaces; (D) spine shows extensive vertebral body compression; (E) pelvis shows a flat acetabulum on both sides, with irregular edges and sclerosis, and poor growth of the femoral epiphyses. Magnetic resonance imaging of the (F) sacroiliac joint shows bilateral sacroiliac arthritis on arthroscopic surface evaluation; (G and H) hip shows inflammatory changes in both hip joints and joint cavity effusion. (I) Whole-body bone scan reveals multiple inflammatory changes.

At presentation, the patient had deformities in the chest, elbows, ankles, and knees, and his metacarpophalangeal, proximal interphalangeal, and distal interphalangeal joints were swollen but not tender. He experienced restricted hip mobility and exhibited difficulty in squatting, limited hip abduction, external rotation, and adduction, and poor spinal prodrome and lateral curvature. The distance between the fingers and the ground was 30 cm, and the occipital wall distance (the distance between the occiput and the wall behind it, when the person is standing upright with the back against the wall) was 0 cm (Fig. 1). Laboratory test results were negative for human leukocyte antigen B27 (HLA-B27), rheumatoid factor, anticyclic citrullinated peptide antibodies, and antinuclear antibodies; erythrocyte sedimentation rate (ESR), C-reactive protein (CRP), and parathyroid hormone levels were normal; and the N-terminal osteocalcin level was 72.92 ng/mL. Radiography revealed joint dysplasia of the elbows, ankles, and metacarpophalangeal, proximal interphalangeal, and distal interphalangeal joints, and significant compression of the vertebrae, which presented as “fish mouth” vertebrae. Magnetic resonance imaging revealed inflammatory changes in the sacroiliac joint and hip, whereas single-photon emission computed tomography revealed inflammatory changes in multiple joints (Fig. 2).

**Figure 3.**
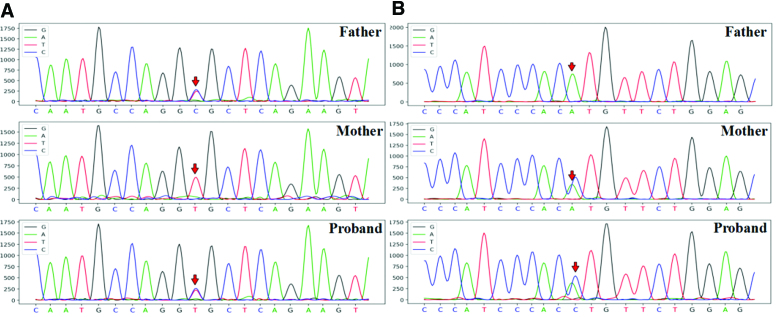
The proband and parents’ mutations. (A) *WISP3*; c.589 + 2T>C. The proband’s father has a heterozygous mutation at chr6: 112386202, and that of the proband’s mother at chr6: 112386202 is wild type. The proband has a heterozygous mutation at chr6: 112386202. (B) *WISP3*; c.721T>G. The proband’s father at chr6: 112389485 is wild type, the proband’s mother has a heterozygous mutation at chr6: 112389485, and the proband has a heterozygous mutation at chr6: 112389485. *WISP3* = *Wnt1-inducible signaling pathway protein 3*.

Whole-exome sequencing of DNA samples from the patient and his parents was performed. Two mutations were found in *WISP3*: c.589 + 2T>C and c.721T>G (Fig. 3). The proband was heterozygous for c.589 + 2T>C, located at chr6: 112386202; the father was heterozygous for the same mutation, and the mother was wild type. Another heterozygous mutation (c.721T>G) was found in *WISP3* at chr6: 112389485, for which the father was wild type, whereas the mother was heterozygous. A compound heterozygous mutation was thus confirmed, consistent with the recessive pattern of PPRD during childhood and the patient’s clinical manifestation. Finally, the patient was diagnosed with PPRD and treated with supplemental calcium, active vitamin D, and glucosamine sulfate. The patient’s joint pain was alleviated; however, joint motion improvement was not obvious after treatment.

## 3. Discussion

We report a novel case of PPRD with sacroiliac and hip arthritis, including bone marrow edema and joint effusion, which contradicts the previous assumption that PPRD is a noninflammatory disease. This case may allow for an improved understanding of PPRD.

PPRD is a rare autosomal recessive disease associated with mutations in *WISP3*. This gene encodes for connective tissue growth factor, which is involved in cell growth and differentiation.^[1,4]^ Its clinical features include articular cartilage dysplasia, arthrocele, platyspondyly, secondary osteoporosis, ankylosis, and joint dysfunction. *WISP3* mutations interfere with chondrocyte stability postnatally, resulting in hyperproliferation of immature chondrocytes and premature degeneration of the articular cartilage, leading to malformations and movement disorders affecting all joints.^[5]^ *WISP3* regulates collagen type II and *SOX9* expression in chondrocytes and affects the expression of aggregation protein.^[6,7]^ PPRD prevalence is approximately one per million, mainly occurring on the eastern coast of the Mediterranean Sea and in the Middle East.^[8]^ Most cases start in childhood, presenting as bilateral protrusion of the proximal interphalangeal joints with progressive involvement of the peripheral joints. Furthermore, approximately one third of cases are accompanied by spinal abnormalities, presenting as short stature and spinal deformity. Early inflammatory changes in the joints are an important factor in developing joint disability.^[9]^

The main clinical manifestations are joint enlargement and movement limitation, with bony enlargement of the interphalangeal joint being a prominent feature. Low back pain during the early stage may lead to a misdiagnosis of spondyloarthritis (SpA). In the late stage, vertebral body shortening and thoracolumbar kyphosis or lateral bending deformity are observed.^[1,9]^ PPRD patients with typical spinal involvement may show ankylosis after hip joint lesions, with difficulty in squatting, pelvic tilt, and claudication in severe cases. Ankle joint changes mainly manifest as joint enlargement. Lumbosacral pain and limited hip mobility of the present patient led to a JIA misdiagnosis. In PPRD patients, other joints, such as the shoulder, wrist, elbow, ankle, and even metatarsophalangeal joints can also develop lesions, mainly characterized by ankylosis and limited mobility but less joint pain.^[1,8]^

The main radiological features of PPRD include cervical bone dysplasia, short and wide femoral neck, enlarged femoral and tibial epiphysis, narrow hip and knee joint space, enlarged metacarpal and phalangeal metaphysis, and osteoporosis (Fig. 3E).^[1,8,9]^ Existing reports suggest that PPRD patients do not usually present with changes in bone erosion or bone marrow edema, typical of JIA. However, the patient presented with typical sacroiliac and hip arthritis, accompanied by bone marrow edema, which was one of the reasons for the misdiagnosis of JIA. The patient’s inflammatory indicators, such as ESR and CRP levels, were normal, and laboratory test results were negative for HLA-B27. The patient’s condition did not improve after previous treatment with sulfasalazine and tumor necrosis factor-α inhibitors. These results suggest that local inflammatory changes, such as bone marrow edema, may occur in PPRD patients but may differ from local joint inflammatory changes seen in both SpA and JIA.^[1,10]^

Laboratory tests for ESR, CRP, rheumatoid factor, antinuclear antibodies, anti-citrullinated protein antibodies, and HLA-B27 were negative, and even the synovial biopsy findings were normal in this patient. Moreover, serum calcium, alkaline phosphatase, growth hormone, thyroid hormone, and insulin levels are usually normal in most PPRD patients,^[1,12]^ as seen in this patient.

There is no effective treatment for PPRD. Current management is supportive and based on pain medications, physical therapy, and surgical intervention. Immunosuppressants such as methotrexate are ineffective, whereas bisphosphonates can improve osteoporosis. Physical therapy may aid in preserving joint mobility.^[13,14]^ In contrast, surgical interventions, such as hip and knee joint replacement, lower-limb realignment, and spinal surgery, are mainly performed in patients with severe joint deformities.^[14]^ Calcitriol, used for osteoporosis, improves the clinical symptoms of PPRD in patients with vitamin D deficiency, suggesting that clinicians should assess vitamin D levels in PPRD patients.^[15]^

The lack of specific clinical and imaging features hinders PPRD diagnosis and can lead to misdiagnosis of JIA, rheumatoid arthritis, or SpA. The final accurate diagnosis depends on whole-exome sequencing.^[16]^ A PPRD diagnosis should be considered for patients presenting with clinical features of unexplained, juvenile onset, multiple cartilage dysplasia, and osseous joints. Genetic testing should be conducted for those with an atypical presentation to avoid misdiagnosis and mistreatment. Familial genetic analysis may be useful for a definitive diagnosis.^[1,3,5]^

PPRD can also be misdiagnosed as JIA, considered the most common form of inflammatory arthritis during childhood, the main difference being that PPRD is a noninflammatory arthropathy.^[17]^ Our case aligns with previously reported cases, with most clinical features consistent with those of PPRD.^[12]^ Moreover, the patient also exhibited inflammatory changes in hip joints and joint effusion, which were not reported in previous cases.^[18]^ Although this case will enrich our understanding of PPRD, more cases are required to elucidate the association between specific PPRD clinical manifestations and genetic variations of *WISP3* and to clarify the particular relationship between PPRD, SpA, and JIA.

## Acknowledgments

We would like to thank Professor Nan Leng for helping to develop the treatment plan, and Guangzhi Xiao, Rong Hua Xie, and Ding Jin for helping to revise the paper. All authors substantially contributed to the study and approved its submission.

## Author contributions

**Conceptualization:** Qing Han, Ronghua Xie.

**Data curation:** Jin Ding.

**Formal analysis:** Junfeng Jia.

**Project administration:** Nan Leng.

**Resources:** Weitao Wang.

**Software:** Weitao Wang.

**Supervision:** Zhaohui Zheng.

**Writing – original draft:** Weitao Wang, Guangzhi Xiao.

**Writing – review & editing:** Weitao Wang, Guangzhi Xiao.

## Supplementary Material


