# Supplementary material for: Progressive pseudorheumatoid dysplasia involving a novel WISP3 mutation and sacroiliac and hip arthritis: A case report and literature review
Source: Medicine (Baltimore). 2023 Jul 7;102(27):e34099. doi: 10.1097/MD.0000000000034099 (PMC10328645; doi:10.1097/MD.0000000000034099)
Supplement: Supplementary file 1 [file medi-102-e34099-s001.pdf]

Supplementary Table S1. Spectrum of *WISP3* mutations and polymorphisms

Location	Variant	Protein change	Reported by	Population
Exon 1	c.43_44delGC	p.Ala15Thrfs*17	Hurvitz et al., 1999	Italy
Intron 1	c.48+dupT	Splicing	Garcia Segarra et al., 2012; Chouery et al., 2012	Jordan, Morocco
	c.49-1G>A	Splicing	Bhavani et al., 2015	India
	c.49-763G>T	p.Phe17Asnfs*42	Garcia Segarra et al., 2012	Belgium
Exon 2	c.105dupT	p.Gly36fs*10	Liu et al., 2015	China
	c.136C>T	p.Gln46*	Ye et al., 2012; Yuet al., 2015	China
	c.156C>A	p.Cys52*	Rai et al., 2016; Temizet al., 2011; Bhavani et al., 2015; Madhuri et al., 2016	Syria, Turkey, Germany, India
	c.168G>T	p.Gln56His	Delague et al., 2005; Garcia Segarra et al., 2012	Lebanon, Belgium
	c.182G>T	p.Cys61Phe	Garcia Segarra et al., 2012	Poland
	c.185delC	p.Pro62Leufs*4	Garcia Segarra et al., 2012	Turkey
	c.190G>A	p.Gly64Arg	Montané et al., 2016	Ecuador
	c.197G>A	p.Ser66Asn	Garcia Segarra et al., 2012; Montané et al., 2016	USA, Italy
	c.232T>C	p.Cys78Arg	Hurvitz et al., 1999	France

	c.233G>A	p.Cys78Tyr	Bhavani et al., 2015; Madhuri et al., 2016	India
	c.236-237CC>AA	p.Ala79Glu	Garcia Segarra et al., 2012	Italy
	c.246delA	p.Glu84Lysfs*21	Hurvitz et al., 1999	Saudi Arabia, Jordan
	c.248G>A	p.Gly83Glu	Delague et al., 2005; Dalal et al., 2012; Ekbote et al., 2013; Rai et al., 2016; Temiz et al., 2011	Lebanon, Syria, India
	c.296A>T	p.Tyr99Phe	Bhavani et al., 2015	India
	c.298T>A	p.Cys100Ser	Bhavani et al., 2015	India
	c.327C>A	p.Tyr109	Garcia Segarra et al., 2012	Turkey
	c.334G>C	p.Gly112Arg	Unpublished data	
	c.340T>C	p.Cys114Arg	Dalal et al., 2012	India
	c.341G>A	p.Cys114Tyr	Yue et al., 2009	China
	c.342T>G	p.Cys114Trp	Liu et al., 2015; Yan et al., 2016; Yu et al., 2015	China
	c.342_343delTG	p.Ala115Ilefs*16	Garcia Segarra et al., 2012	Turkey
Intron 2	c.346+1G>T	p.Tyr109Met195delins9	Garcia Segarra et al., 2012	Turkey
	c.347-2A>G	p.Tyr109Met195delins9	Bhavani et al., 2015	India
	c. 347-1_347-3delCAG	Splicing	Bhavani et al., 2015	India
Exon 3	c.348C>A	p.Tyr116*	Madhuri et al., 2016	India
	c. 396T>G	p.Cys132Trp	Yan et al et al., 2016	China

	c.433T>C	p.Cys145Arg	Dalal et al., 2012; Bhavani et al., 2015	India
	c.434G>A	p.Cys145Tyr	Hurvitz et al., 1999; Garcia Segarra et al., 2012	Italy
	c.530C>A	p.Ser177*	Bhavani et al., 2015	India
	c.536_537delGT	p.Cys179*	Delague et al., 2005	Syria
	c.589G>C	p.Ala197Glyfs*5	Delague et al., 2005	Syria
	c.589G>A	p.Ala197Glyfs*5	Garcia Segarra et al., 2012	Turkey
	c.589+1G>A	Splicing: IVS4 ds +1	Rai et al., 2016	India
Intron 3	c.589+27C>G	p.Ala197Glyfs*5	Garcia Segarra et al., 2012	Italy
	c.589+2T>C	splicing	Sun et al., 2012	China
Exon 4	c. 594_598delATAGA	p.Tyr198*	Madhuri et al., 2016.	India
	c.621_622delAAinsT	p.Lys207Asnfs*25	Garcia Segarra et al., 2012	USA
	c.624_625insA	p.Cys209Metfs∗21	Ye et al., 2012	China
	c.624delA	p.Lys208fs∗24	Liu et al., 2015	China
	c.624dupA	p.Cys209Metfs*21	Ye et al., 2012; Garcia Segarra et al., 2012; Bhavani et al., 2015; Chouery et al., 2017	China, Turkey, India, Tunisia
	c.625dupT	p.Cys209Leufs∗21	Yan et al., 2016	China
	c.643+1G>A	Not known*	Rai et al., 2016	India

c.667T>G	p.Cys223Gly	Ye et al., 2012; Luo et al., 2015; Yan et al., 2016; Yu et al., 2015; Al Kaissi et al., 2017	China, Russia
c.670G>A	p.Gly224Arg	Garcia Segarra et al., 2012	Italy
c. 670dupA		Hu et al., 2017	China
c.677G>T	p.Gly226Val	Garcia Segarra et al., 2012; Madhuri et al., 2016	UK, India
c.679dup	p.Cys227Leufs*21	Ye et al., 2012; Yan et al., 2016	China
c.682T>C	p.Ser228Pro	Dalal et al., 2012; Ekbote et al., 2013	India
c.683_684insT	p.Asn229*	Bhavani et al., 2015	India
c.685_686insATCTA	p.Arg230Leufs*4	Bhavani et al., 2015	India
c.708dupC	p.Asn237Glnfs*3	Garcia Segarra et al., 2012	Turkey
c.716_722del	p.Glu239fs*16	Sun et al., 2012	China
c.721T>G	p.Cys241Gly	Yan et al., 2016	China
c.719_725delTGAGAAA		Sun et al., 2012	China
c.725_726delAA	p.Lys242Argfs*36	Garcia Segarra et al., 2012	Italy
c.727_731delGAGAA	p.Glu243Lysfs*34	Garcia Segarra et al., 2012	Turkey
c.729-735delGAGAAAA	p.Glu243Aspfs*13	Ye et al., 2012	China
c.739_740delTG	p.Cys247Leufs*31	Ehl et al., 2004; Dalal et al., 2012; Bhavani et al., 2015	Germany, India
c.740_741delGT	p.Cys247Leufs*31	Bhavani et al., 2015	India

	c.756C>A	p.Cys252*	Luo et al., 2015; Hu et al., 2017	China
	c.779_783+1delTAAAGG	p.Ile260Asnfs*17	Bhavani et al., 2015	India
Exon 5	c.794_795delGT	p.Cys265LeufsX31	Cassa et al., 2016	Pakistan
	c.802T>G	p.Cys268Gly	Dalal et al., 2012	India
	c.804delC	p.Gln269Asnfs*44	Bhavani et al., 2015	India
	c.805delC	p.Q269Nfs∗44	Bhavani et al., 2015	India
	c.807A>G	p.Gln269Gln	Hurvitz et al., 1999; Garcia Segarra et al., 2012	Jordan, Turkey
	c.840delT	p.Phe280Leufs*33	Sun et al., 2012; Yang et al., 2013	China
	c.850G>T	p.Gly284*	Garcia Segarra et al., 2012	Turkey
	c.857C>G	p.Ser286*	Garcia Segarra et al., 2012; Yu et al., 2015	Turkey, China
	c.862_863dupAC	p.Gln289Leufs*25	Hurvitz et al., 19999; Garcia Segarra et al., 2012	USA, Turkey
	c.866dupA	p.Ser290Glufs*13	Ye et al., 2012; Garcia Segarra et al., 2012; Yu et al., 2015	China, Turkey
	c.866_867insA	p.Gln289fs∗31	Sun et al., 2012; Ye et al., 2012	China
	c.868-869delAG	p.Ser290Leufs*12	Hurvitz et al., 1999; Garcia Segarra et al., 2012	Iran, Italy
	c.947_951delAATTT	p.Gln316Argfs*5	Dalal et al., 2012	India
	c.993G>A	p.Trp331*	Hurvitz et al., 1999	Italy

	c.1000T>C	p.Ser334Pro	Sun et al., 2012	China
	c.1004G>A	p.Cys335Tyr	Garcia Segarra et al., 2012	Italy
	c.1010G>A	p.Cys337Tyr	Ekbote, 2013; Bhavani et al., 2015; Madhuriet al., 2016	India
	c.1013A>T	p.Gln338Leu	Nakamura et al., 2007	Japan
